# Drug delivery systems: advanced technologies potentially applicable in personalised treatment, educational measures

**DOI:** 10.1186/1878-5085-5-S1-A15

**Published:** 2014-02-11

**Authors:** Jorge FJ Coelho

**Affiliations:** 1 Department of Chemical Engineering, University of Coimbra, Portugal

## 

This seminar intends to present a book recently published that is part of a series dedicated to recent advances on preventive, predictive and personalised medicine (PPPM). It is mainly focused on the theme of “Drug delivery systems: advanced technologies potentially applicable in personalised treatments”. The critical topics involving the development and preparation of effective drug delivery systems, such as: polymers available, self-assembly, nanotechnology, pharmaceutical formulations, three dimensional structures, molecular modelling, tailor-made solutions and technological tendencies, are carefully discussed. The understanding of these areas constitutes a paramount route to establish personalised and effective solutions for specific diseases and individuals. This book was organised to constitute an important reference for didactic purposes as well as to provide the state-of-art on the area of DDS applicable in personalised treatments.

